# Commentary: What Is the Link between Stringent Response, Endoribonuclease Encoding Type II Toxin-Antitoxin Systems and Persistence?

**DOI:** 10.3389/fmicb.2017.00191

**Published:** 2017-02-14

**Authors:** Laurence Van Melderen, Thomas K. Wood

**Affiliations:** ^1^Laboratoire de Génétique et Physiologie Bactérienne, Faculté des Sciences, Université Libre de Bruxelles (ULB)Gosselies, Belgium; ^2^Department of Chemical Engineering, Pennsylvania State UniversityState College, PA, USA

**Keywords:** toxin-antitoxin, *lon* protease, persistence, ppGpp, poly-phosphate

In a recent paper published in Frontiers in Microbiology, Ramisetty et al. (2016) questioned the mainstream model regarding bacterial persistence proposed by the group of K. Gerdes (Maisonneuve et al., 2011, 2013). Persistence is an important phenomenon thought to contribute to infectious diseases chronicity and antibiotic resistance selection (Michiels et al., 2016). This reversible and low frequency phenotypic switch allows bacteria to enter a particular physiological state in which they can sustain the presence of a given antibiotic (Kaldalu et al., 2016; Wood, 2016). It is commonly thought that persister cells are “dormant” i.e., non-replicating and metabolically inactive. Therefore, the idea that toxin-antitoxin systems (TAs) could be involved in persistence was quite tempting since toxins from type II TAs are cell growth inhibitors (Hayes and Van Melderen, 2011). To test this appealing hypothesis, Maisonneuve et al. deleted 10 systems comprising endoribonucleases as toxins in the *E. coli* lab strain (note this is only about 25% of the known *E. coli* TAs) and presented results that indicate that the resulting Δ10 strain was strongly affected for persistence upon treatment with ciprofloxacin or ampicillin (Maisonneuve et al., 2011). This was not attributable to deletion of any specific system. On the contrary, successive deletion of systems (any among the 10 systems studied) progressively diminished persistence frequency, showing that TAs are redundant and have a cumulative effect. Moreover, testing the persistence frequency in Δ*lon*, Δ*relA*Δ*spoT*, and Δ*ppk-ppx* mutants, the authors claimed that persistence is also dependent on the Lon protease, on guanosine tetraphosphate (ppGpp) and polyphosphate (polyP) (Maisonneuve et al., 2013). The proposed model is that under stress conditions, ppGpp concentration increases thereby inhibiting polyphosphatase (Ppx). As a result, polyP accumulates, activates Lon, which in turn degrades efficiently the antitoxins from the 10 TAs. Toxins are then liberated and able to degrade bulk mRNAs leading to translation inhibition and persistence.

While Ramisetty et al. actually confirmed that the Δ10 strain is less persistent than the wild-type strain when treated with ciprofloxacin or ampicillin (Ramisetty et al., 2016), they also showed that the Δ10 strain presents a reduced minimal inhibitory concentration (MIC) to ampicillin and ciprofloxacin, showing that this strain presents intrinsic susceptibility to these antibiotics. Note that on the contrary to resistance, MIC should not influence persistence (Brauner et al., 2016). The Δ10 strain also appears to be affected for growth at least in the conditions tested (LB medium, 37°C). Maximum growth rate and CFU/ml after 12 h of growth are significantly lower as well as the capacity to form biofilms. Altogether, these data show that the Δ10 strain is less fit than the wild-type strain. The authors proposed that deletion of TAs might have a polar effect and thereby affect downstream gene expression and strain fitness. To confirm this, whole genome sequencing as well as gene expression analysis should be performed. One should also be careful with approaches that involve multiple deletions. Genome manipulation using the λRed system and counter-selection methods based on *parE* toxin expression might have generated multiple genomic rearrangements and therefore lead to a fitness decrease.

Other groups investigated the implication of the 10 TAs as well as that of Lon, ppGpp, and polyP in persistence to various classes of antibiotics. While the Δ10 strain was less persistent upon gentamycin treatment (contrary to what is reported in Ramisetty et al., 2016), the Δ*lon*, Δ*relA*Δ*spoT*, and Δ*ppk* mutants were not affected (Shan et al., 2015). Moreover, β-lactam treatment did not affect persistence of a Δ*lon* mutant (Theodore et al., 2013; Chowdhury et al., 2016), on the contrary to what was previously reported by the Gerdes lab (Maisonneuve et al., 2011). Finally, for ciprofloxacin, persistence of the Δ*lon* strain was strongly affected but the decrease in persistence was solely dependent on SulA, a SOS cell division inhibitor and Lon substrate, since the double Δ*lon*Δ*sulA* mutant presents similar persister frequency to that of the wild-type strain (Theodore et al., 2013). It is well described that Lon mutants are unable to recover from an SOS-inducing treatment, such as ciprofloxacin, due to SulA accumulation and incapacity of cell division to resume (Mizusawa and Gottesman, 1983; Schoemaker et al., 1984).

This body of data from different groups casts serious doubts about the polyP-dependent TAs regulation model. Strikingly, this model implies that the Lon protease degrades the antitoxins of interest in a polyP-dependent manner and at comparable rates. While most antitoxins appear to be indeed susceptible to Lon (Christensen et al., 2001, 2003, 2004; Jørgensen et al., 2009; Prysak et al., 2009; Christensen-Dalsgaard et al., 2010), at least four of them (MazE, DinJ, MqsR, and HigA) have been described to be also susceptible to Clp proteases (Prysak et al., 2009; Christensen-Dalsgaard et al., 2010), indicating that Lon might not be solely responsible for these TAs activation. Moreover, for most antitoxins, polyP-dependency was not tested. Ramisetty et al. have probed YefM (antitoxin from one of the 10 systems of interest) degradation using primer extension as a proxy (Ramisetty et al., 2016). This method relies on the fact that TAs are autoregulated at the level of transcription by a complex formed of antitoxin and toxin proteins (Hayes and Van Melderen, 2011). It is commonly thought that upon antitoxin degradation, transcriptional activation of the cognate TA operon is observed and can be detected by northern blots and/or primer extension. The data obtained by this method indicate that YefM is indeed degraded by Lon but in a ppGpp and polyP-independent manner, in contrast to what was reported by the Gerdes group (Maisonneuve et al., 2011, 2013). Moreover, using overexpression of Lon as way to probe TAs activation, Van Melderen's lab in collaboration with Gerdes lab showed that the *yefM-yoeB* TAs appears to be the only system producing mRNA cleavage (Christensen et al., 2004). These data were confirmed by Ramisetty et al. (2016). Critically, Ramisetty et al. showed that mRNA cleavage by YoeB during Lon production is still observed in a Δ*ppk-ppx* strain, thereby confirming that polyP is not required to activate the *yefM-yoeB* system (Ramisetty et al., 2016). Also, *in vitro*, polyP deactivates Lon rather than activating it (Osbourne et al., 2014).

Still regarding antitoxin degradation, surprising discrepancies between papers of Maisonneuve et al. (2011, 2013) and previous works of the Gerdes group were noted by Ramisetty et al. (2016). In papers published in 2001 (Christensen et al., 2001) and 2003 (Christensen et al., 2003), the Gerdes group clearly demonstrated using northern blot, primer extension and/or Western blot analyses that degradation of RelB and MazE (antitoxins from the 10 systems of interest) is Lon-dependent but independent of ppGpp. However, according to the current model proposed by the same group, degradation of these antitoxins requires ppGpp and polyP. The reasons for these discrepancies were never discussed in the concerned papers.

As a conclusion, the link between the stringent response, endoribonuclease encoding type II toxin-antitoxin systems and persistence appears to be weak. Type II endoribonucleases toxins and ppGpp are clearly not the only players for generating persister cells. Multiple reports indicate that other molecular mechanisms are involved in persister cell formation such as type II toxins with cell wall synthesis inhibition activity or type I toxins as well as the SOS response (Lioy et al., 2012; Kaldalu et al., 2016; Wood, 2016). In addition to antitoxin degradation, the polyP-dependent TA regulation model raises important unanswered questions, among them: (1) what is the expression level of the 10 different TAs in steady-state (repression) and activation (derepression) conditions, (2) how is Lon activated to degrade antitoxins in toxin-antitoxin complexes, (3) what are the kinetics of toxin release from antitoxin-toxin complexes, and (4) what is the affinity of toxins for their respective targets. In addition, the upstream part of the model is also surprisingly little-documented, notably how polyP affects Lon substrate degradation (if at all), and how ppGpp is affecting persistence. For example, it was recently shown that persister cells can form in the absence of ppGpp, although to a lower level (Chowdhury et al., 2016). Hence, how persister cells form in clinically-relevant settings and whether persister cells are chiefly responsible for antibiotic failure remains to be elucidated.

Knowing that TAs are very diverse and abundant in prokaryotic genomes, that they are part of the accessory genome and they move by horizontal gene transfer (Anantharaman and Aravind, 2003; Makarova et al., 2009; Guglielmini and Van Melderen, 2011; Leplae et al., 2011; Ramisetty and Santhosh, 2016), trying to squeeze them all in the same functional model is very reductive. We believe that these small, evolutionary-successful systems deserve better consideration!

## Author contributions

All authors listed, have made substantial, direct and intellectual contribution to the work, and approved it for publication.

### Conflict of interest statement

The authors declare that the research was conducted in the absence of any commercial or financial relationships that could be construed as a potential conflict of interest.
